# Study on the reaction strategy of directional alkylation fulfilled by controlling the adsorption pose of benzene and methanol in space with Ru/HZSM-5

**DOI:** 10.1186/s13065-021-00761-2

**Published:** 2021-05-21

**Authors:** Dongliang Wang, Yanwei Li, Yu Zhao, Dong Ji, Peng Dong, Guixian Li

**Affiliations:** College of Petrochemical Technology, Lanzhou University of Technology, Lanzhou, 730050 People’s Republic of China

**Keywords:** Benzene alkylation, MTO side reactions, Ru/HZSM-5, Catalyst stability

## Abstract

**Background:**

The alkylation of benzene with methanol is an important synthetic method that is widely used in various chemical production processes. However, obtaining a high selectivity for xylene and annihilating the MTO side reaction remain challenges.

**Results:**

In this work, a Ru/HZSM-5 catalyst was prepared using ZSM-5 as the precursor by a chemical precipitation method. Both XRD and TEM confirmed that Ru nanoparticles were evenly dispersed on the surface of the ZSM-5. The catalytic performance of benzene alkylation with methanol on the Ru/HZSM-5 catalyst was investigated. The results showed that the Ru/HZSM-5 catalyst could completely annihilate the MTO side reaction with a high conversion efficiency of benzene and selectivity for xylene, which resulted from the large gap between the transgression energy value of Ru and the ionization potential value of benzene, and the acidity of the catalyst changed greatly.

**Conclusion:**

These findings may offer not only a new and efficient multifunctional catalyst for benzene alkylation but also fundamental insight into the catalytic mechanism of the Ru/HZSM-5 catalyst.

## Introduction

Toluene and xylene are important chemical intermediates for the production of fine chemicals, and have been widely used in the chemical industry [1–5]. Combined with China’s resource allocation, both benzene and methanol are present in excess or even waste in the petrochemical and coal chemical industries, respectively. Therefore, high added value would be obtained by the production of toluene and xylene by alkylation of benzene with methanol; this is of great significance. This method would facilitate the future coupled development of the petrochemical industry and coal chemical industry and produce a new situation. However, the key difficulties for benzene alkylation with methanol have been the MTO side reaction, which results in deactivation of the catalyst. These drawbacks would greatly hinder the alkylation reaction for industrial production.

The MTO side reaction and carbon deposition by screening or modifying catalysts have been suppressed by numerous efforts. For example, the Hu team researched modulation of the ratio of silicon to aluminum of the catalyst and found that it could suppress the formation of ethylbenzene [6]. Much research has been done by changing the acidity of zeolite catalysts. SiO_2_, P_2_O_5_, MgO [7], different acids, and different bases [8] were used to regulate the amount or type of acid (strong acid or weak acid, L acid or B acid). Because an acid catalyst was used for the benzene alkylation reaction, the acid amount of the catalyst determined the reaction path and the amount of target product [9, 10]. New research showed that the byproduct ethylbenzene was suppressed over a Pt/ZSM-5 catalyst in the alkylation of benzene with methanol [11]. However, the MTO side reaction was not annihilated in this reaction in a hydrogen atmosphere, with alkenes being hydrogenated to form alkanes. Therefore, the number of side reactions has not decreased, which would result in the waste of raw materials.

In the present work, we synthesized a Ru/HZSM-5 catalyst with Ru nanoparticles using a chemical precipitation method. The structure and morphology as well as the thermal stability were characterized using XRD, EDS, TEM, FT-IR and TGA. The catalytic performance for the alkylation of benzene with methanol over the Ru/HZSM-5 catalyst showed that the Ru/HZSM-5 catalyst could completely annihilate the MTO side reaction with a high conversion efficiency of benzene and selectivity for xylene. To the best of our knowledge, a feasibility study of Ru nanoparticle-loaded ZSM-5 catalysts for annihilating the MTO side reaction has rarely been reported and may provide fundamental guidance for the alkylation of benzene with methanol.

## Experimental

### Preparation of catalysts

ZSM-5 as a precursor was purchased from Lanzhou Jia Te Xing Co., Ltd. (China) and then roasted at 773 K for 4 h. A certain amount of ZSM-5 and ammonium carbonate were mixed in a three-necked flask at room temperature for 4 h. Then, RuCl_3_·3H_2_O was added to the three-mouth bottle dropwise at room temperature and stirred for 20 h. The stirred mixture was filtered and washed. Then, more importantly, the sample was dried at 383 K for 24 h to obtain Ru_2_(CO_3_)_3_/HZSM-5. The Ru_2_(CO_3_)_3_/HZSM-5 then was roasted at 773 K for 4 h to obtain Ru_2_O_3_/HZSM-5. Ru_2_O_3_/HZSM-5 was reduced in a H_2_ environment by employing a fixed-bed reactor at 773 K for 3 h to obtain Ru/HZSM-5 (4 wt. %). In summary, a catalyst of H-form and Ru-loaded (Ru/HZSM-5) synthesis was synthesized at one time.

### Catalyst characterization

The samples were characterized using XRD, EDS, TEM and FT–IR. The equipment and test parameters used are the same as reported in our prior work [12]. TGA analysis was performed on a Perkin-Elmer TG/DTA-6300 thermal analyzer. Pyridine adsorption measurements were performed by infrared spectroscopy aiming to determine the nature of acidic sites (Brönsted and Lewis).

### Catalytic activity evaluations

The catalyst activity was evaluated by alkylation of benzene with methanol, where the operating procedures and equipment used were the same as those in our previous studies [12]. The difference was that the catalyst was Ru/HZSM-5, and the collected products were analyzed by a Fuli GC9790.

## Results and discussion

### Catalyst characterizations

**Fig. 1:**
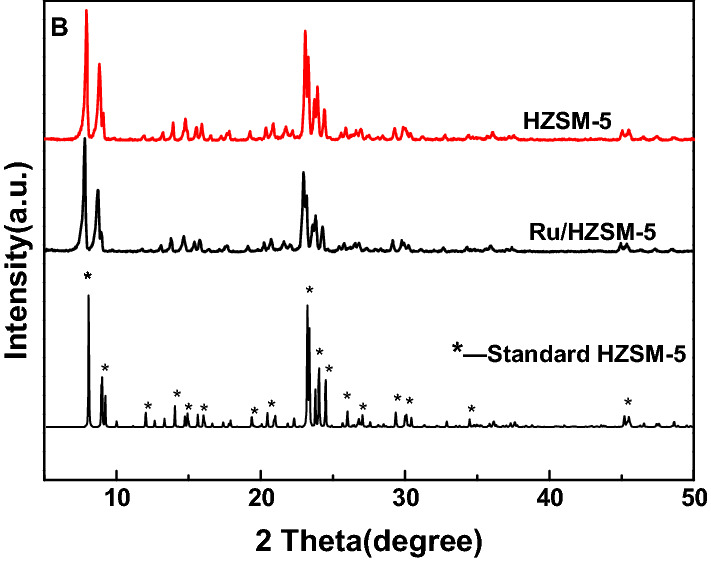
XRD patterns of HZSM-5 and Ru/HZSM-5 catalysts

**Fig. 2:**
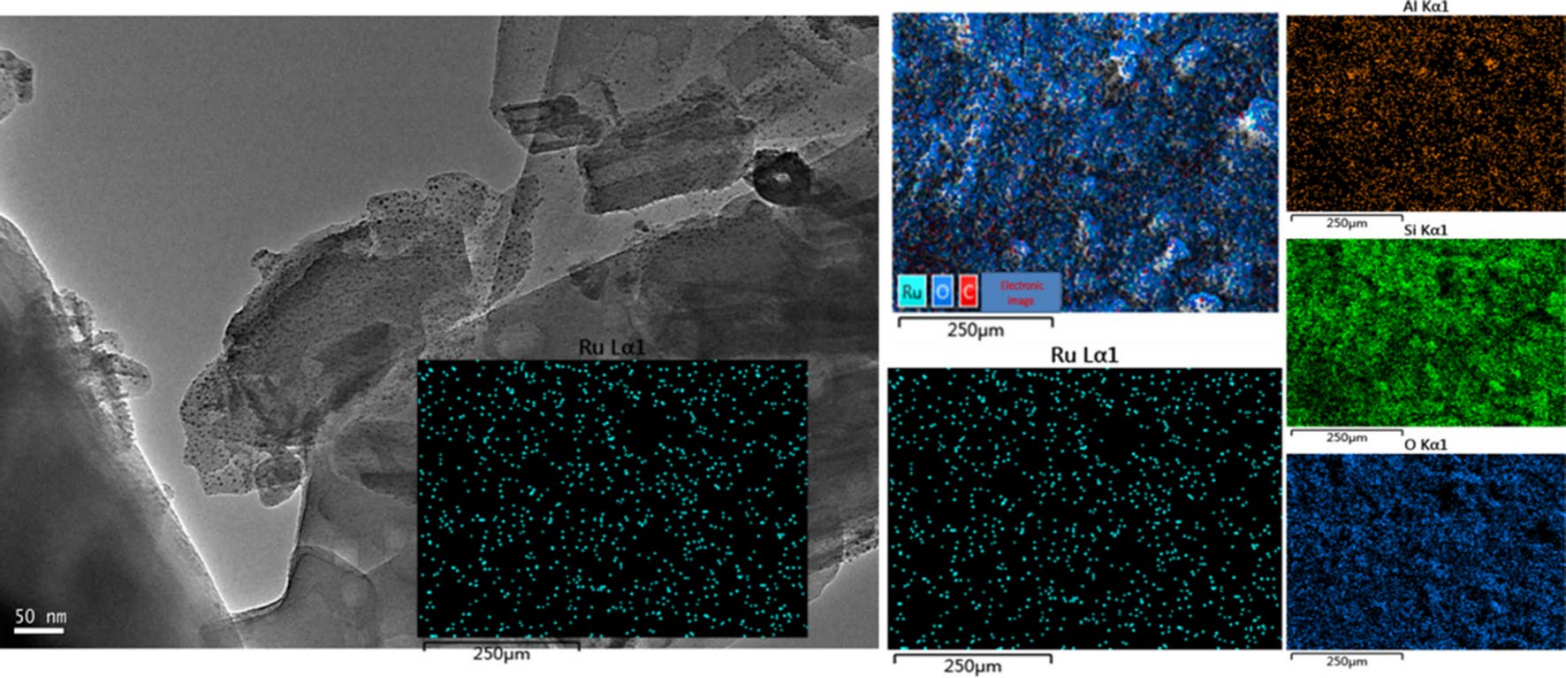
TEM image of Ru/HZSM-5 and EDS mapping of Ru/HZSM-5

Figure 1 shows the XRD characterization results of the HZSM-5 and Ru/HZSM-5 catalysts. Both HZSM-5 and Ru/HZSM-5 exhibited well-resolved diffraction peaks at 2θ = 8° ~ 10° and 20° ~ 25°, which were attributable to the MFI structure [13–15], indicating the retention of the zeolite structure in Ru/HZSM-5 after treatment. Nevertheless, the height of the characteristic peaks for Ru/HZSM-5 was decreased, which could result from desilicification and suggested a decrease in crystallinity. In addition, the characteristic peak of Ru particles was not observed, indicating the good dispersion of Ru particles on the surface of ZSM-5, which could also be confirmed by EDS mapping (Fig. 2). The high-scatter photos in Fig. 2 show of Ru/HZSM-5 obtained by TEM and EDS mapping of Ru/HZSM-5, which exhibited an irregular and flat structure with smooth surfaces and angular edges. It was clear that the Ru nanoparticles were dispersed evenly on the surface of HZSM-5, in accordance with the XRD and EDS results.

**Fig. 3:**
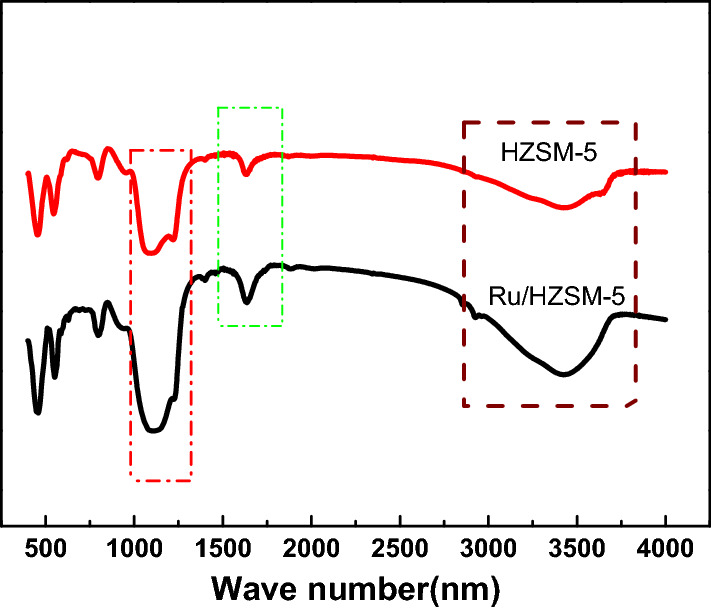
FT-IR spectra of the HZSM-5 and Ru/HZSM-5 catalysts

Figure 3 shows the FTIR spectra of HZSM-5 and Ru/HZSM-5. The vibration strength of the skeletons of HZSM-5 and Ru/HZSM-5 did not change much. However, compared with HZSM-5, the intensity of the (Si/Al)–OH tetrahedron at 1111 cm^−1^ was slightly increased, and the stretching vibrations of –OH groups at 1634 cm^−1^ and 3420 cm^−1^ were also increased.

**Fig. 4:**
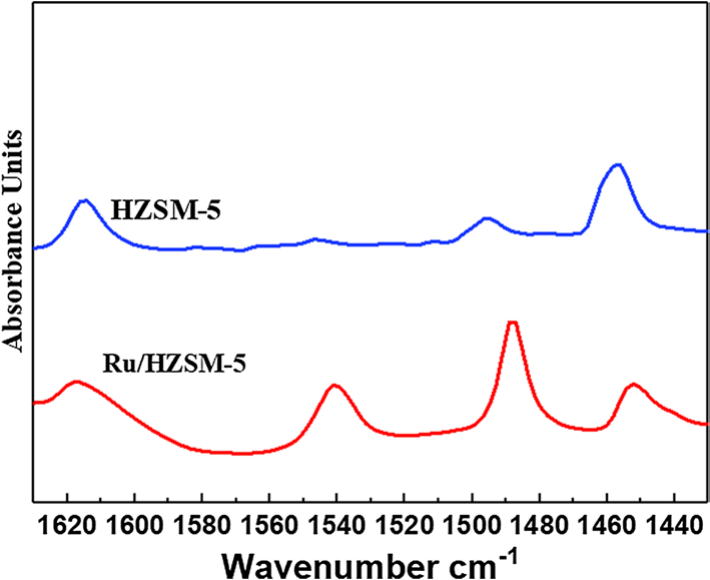
IR spectra of the samples after pyridine desorption

The acidity of HZSM-5 and Ru/HZSM-5 catalysts was investigated by Py-IR spectroscopy in Fig. 4. The spectra exhibits four bands at frequencies 1620, 1540, 1490 and 1450 cm^−1^ can be detected on these samples. The strong absorption bands at 1620 cm^−1^ and at 1450 cm^−1^ indicate the presence of coordinated pyridine at the Lewis acid sites of the catalyst [16]. IR band appearing at 1490 cm^−1^ is associated to the vibration of the pyridinic ring on Brönsted and Lewis acid sites [17]. In addition, IR band near 1540 cm^−1^ is due to the adsorption of pyridine coordinated on Brönsted acid sites [17].

Figure 4 shows Py-IR spectra of these catalysts at specific temperature. The amount of acid sites increased in the sequence: HZSM-5 < Ru/HZSM-5. The main reasons were that there was an ion exchange process in the preparation process of the catalyst, and the introduction of Ru would increase the active site of B acid on the surface of the catalyst, making the active site an acid environment system with easy adsorption and activation of benzene molecules. Based on above indicated that: the quantity of Brönsted acid sites was good for enhancing the conversion of benzene and the activity of catalysts [18], which was derived from the active site of B acid formed by the introduction of Ru.

### Ru/HZSM-5 catalysts for annihilating the MTO side reaction in benzene alkylation with methanol

Currently, there are many byproducts in the alkylation process of benzene, which are attributed to the side reaction of methanol in benzene alkylation. For instance, ethylbenzene, carbon deposition, etc. were all due to olefins [11]. Benzene alkylation with methanol was carried out over the HZSM-5 and Ru/HZSM-5 catalysts. The results are summarized in Table 1. The conversion of benzene was 48.3%, and the selectivity of xylene was 18.0% over Ru/HZSM-5, which values were nearly the same as those of the HZSM-5 catalyst (47.6 and 18.1%, respectively), indicating that the loading of Ru nanoparticles in HZSM-5 had no adverse effect on the conversion efficiency of benzene and selectivity for xylene. However, the main byproducts, such as ethylbenzene and trimethylbenzene, were enormously suppressed. It is well known that ethylbenzene originates from the reaction of benzene and ethylene. The selectivity of ethylbenzene dropped from 1.3% (for HZSM-5) to almost zero (for Ru/HZSM-5) due to the great decrease in ethylene after the introduction of Ru nanoparticles, which was also confirmed by the Fuli GC9790.

**Table 1:** Product content of benzene alkylation with methanol over different catalysts

Catalyst	Benzene conversion/%	Product selectivity/%				
		Toluene	Xylene	Ethylbenzene	Trimethylbenzene	Others
HZSM-5	47.6	76.3	18.1	1.3	2.1	2.2
Ru/HZSM-5	48.3	79.2	18.0	0	0.9	1.9

Reaction conditions: catalyst = 0.5 g, T = 673 K, reaction time = 3 h, pressure = normal atmosphere, feed (B/M) ratio = 1:1, with a cofeed of N_2_

As seen in Table 2, the composition of gas phase products was totally different over the Ru/HZSM-5 catalyst and HZSM-5 catalyst in the alkylation reaction. It can be seen from the analysis that methanol would participate completely in the reaction and form gaseous substances (mainly CH_2_=CH_2_ and CH_2_=CH_2_–CH_3_) over the HZSM-5 catalyst. Therefore, more ethylbenzene byproducts were easily formed by the HZSM-5 catalyst. For the Ru/HZSM-5 catalyst, ethylene and propylene were not detected in the gas phase products; however, the content of methanol was relatively high, which suggested that the Ru nanoparticles suppressed the formation of ethylbenzene as a result of the MTO reaction being annihilated by the Ru/HZSM-5 catalyst.

**Table 2:** Gas phase composition of HZSM-5 and Ru/HZSM-5

Catalyst	Gas phase				
	Methane	Methanol	Ethylene	Propylene	Others
HZSM-5	+	–	++	++	++
Ru/HZSM-5	+	++	–	–	+

Reaction conditions: catalyst = 0.5 g, T = 673 K, reaction time = 3 h, pressure = normal atmosphere, feed (B/M) ratio = 1:1, with a cofeed of N_2_. Explanation:  + means very little;  ++ means more; –means none

**Fig. 5:**
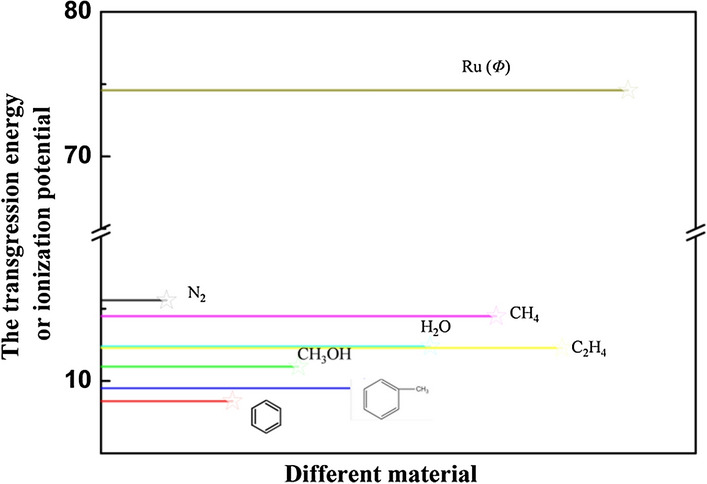
Ru transgression energy and ionization potential of different material [19]

**Fig. 6:**
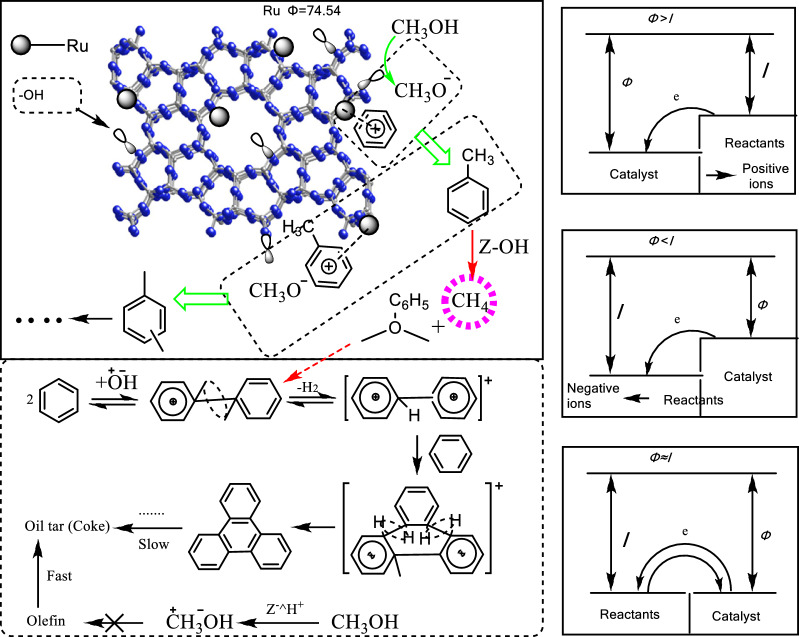
Chemical adsorption electron transfer and adsorption state, mechanism of catalytic reaction, *Φ* ~ transgression energy of metal; *I* ~ ionization potential of reactant molecules

The mechanism of Ru/HZSM-5 was discussed. It is well known that the adsorption process plays a key role in the catalytic reaction process. Therefore, the adsorption of reactants was the most important step of the catalytic processes, during which the adsorption center on the surface of metal catalysts (Ru) and the reactant molecules (benzene or methanol) must form bonds with electron transfer or electron sharing to produce chemical adsorption. The nature of the chemical bonds depends on the electron layer structure of the metal and the physical–chemical properties of the reactants, corresponding to the transgression energy of the metal and ionization potential of the chemical molecules. According to relevant data and theories [19], the larger the difference between the transgression energy (*Φ*) of metal and ionization potential (*I*) of the reactant molecules, the easier it is for them to be adsorbed and activated. The transgression energy value of Ru is 74.5 eV. The ionization potential values of benzene, toluene and methanol, ethylene, water, methane and nitrogen were 8.5, 9.4, 10.9, 12.2, 12.3, 14.5, and 15.5 eV, respectively (Fig. 5). Comparing the transgression energy of Ru with the ionization potential of chemical molecules, the electron transfer direction of the reactant and the adsorption state are different in the process of chemical adsorption (Fig. 6). For the study of the alkylation reaction over the Ru-acid bifunctional catalyst used in this paper, benzene was one of the most easily adsorbed species because of the largest difference between the transgression energy of Ru and the ionization potential of benzene. Once the positive ion of benzene is formed, it could reduce the electron work function of the catalyst surface and promote the catalytic process on Ru/HZSM-5.

On the surface of the catalyst, benzene was adsorbed easily by the active center of Ru metal, and methanol was adsorbed easily by the active center of the acid. Moreover, nitrogen was used as the transport medium to form the optimal catalytic environment for the alkylation of benzene and methanol. When benzene and methanol were activated and mutually attracted, toluene and xylene were generated. Since the ionization potential of toluene was higher than that of benzene, it was easy for products to detach from the catalyst surface and thus they could not react any further. Therefore, the use of the Ru/HZSM-5 catalyst could effectively reduce the amount of trimethylbenzene and other byproducts.

### Catalyst stability

**Fig. 7:**
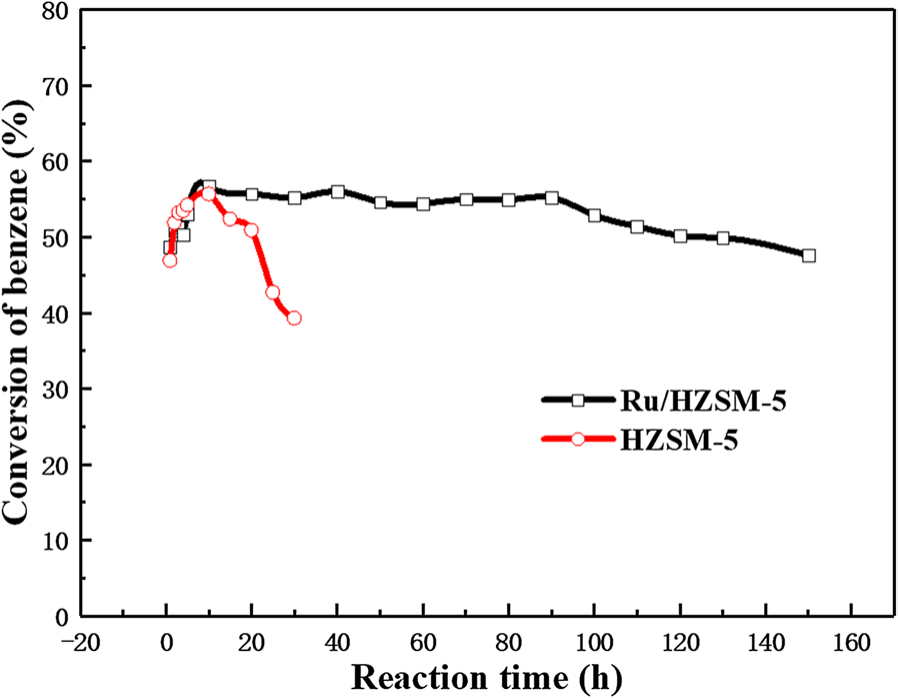
Stability of HZSM-5 and Ru/HZSM-5 in benzene alkylation with methanol

**Fig. 8:**
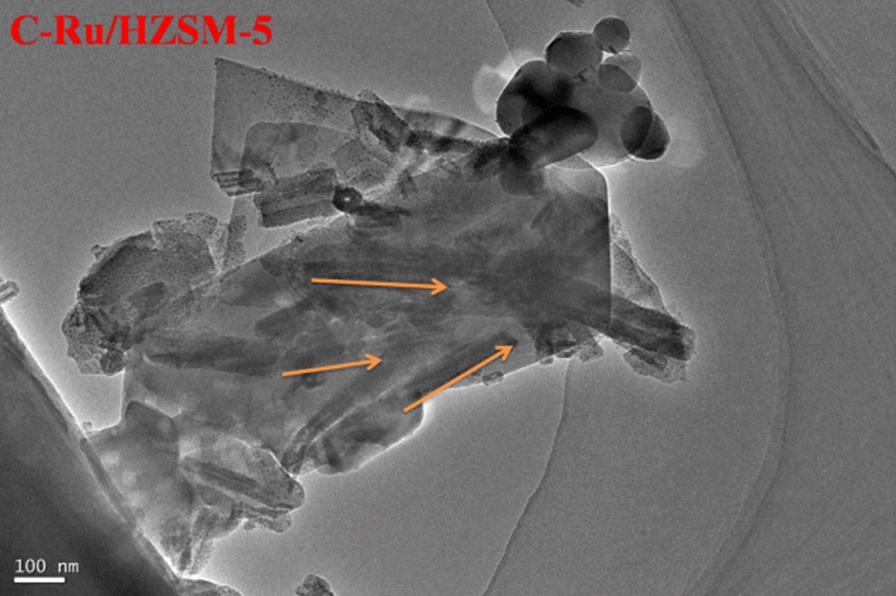
TEM image of C-Ru/HZSM-5

The stability of the catalysts was another important factor in evaluating the performance during practical usage in specific applications. The experimental results show that the thermal stabilities of HZSM-5 and Ru/HZSM-5 were very different, as shown in Fig. 7. After 150 h, Ru/HZSM-5 retained a conversion of benzene of 50%, which was higher than that of HZSM-5 (20 h), suggesting its excellent stability [20]. From the TEM image (Fig. 8), black mulch is discovered in Ru/HZSM-5 after the reaction (written C-Ru/HZSM-5 hereafter), which contributes to carbon particles. The coke of catalysts can be divided into filament mold (soft coke) and graphite mold coke (hard coke).

**Fig. 9:**
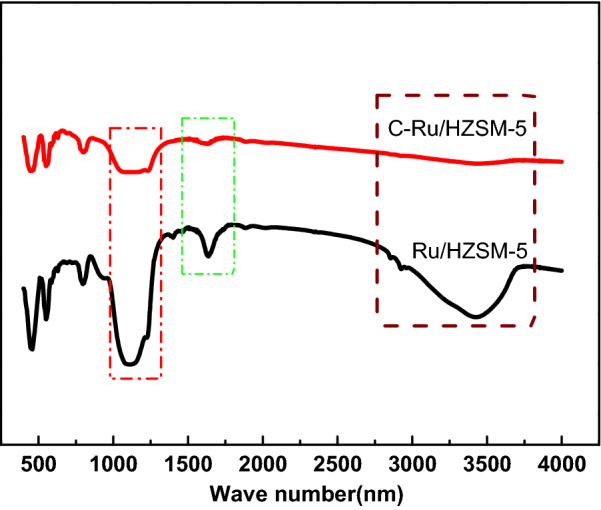
FT-IR spectra of the Ru/HZSM-5 and C-Ru/HZSM-5 catalysts

The FTIR spectra of Ru/HZSM-5 and C-Ru/HZSM-5 are shown in Fig. 9. The vibration strength of the skeletons of Ru/HZSM-5 and C-Ru/HZSM-5 changed considerably. The change shown by C-Ru/HZSM-5 was that the intensity of the Si/Al–OH tetrahedron at 1111 cm^−1^ was decreased, and the stretching vibrations of the –OH groups at 1634 cm^−1^ and 3420 cm^−1^ either disappeared or were drastically decreased. The reason for such a large change was the carbon deposition on Ru/HZSM-5 catalysts.

**Fig. 10:**
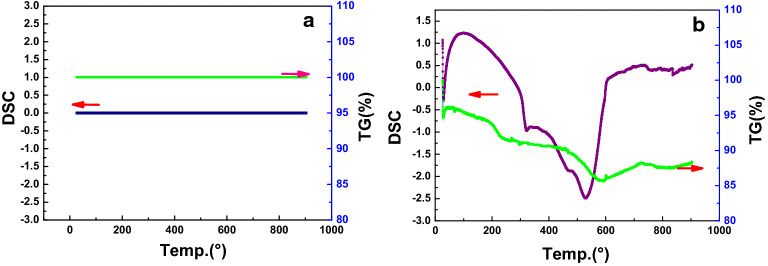
TG-DSC characterization of Ru/HZSM-5 **a** and C-Ru/HZSM-5 **b**

TGA was used to investigate the thermal stability of the Ru/HZSM-5 catalyst after usage. Figure 10 shows the TG-DSC curve of Ru/HZSM-5 catalyst. As shown in Fig. 10, the weight of the Ru/HZSM-5 catalyst barely changed in the temperature range of the TG-DSC test, indicating the excellent thermal stability of the Ru/HZSM-5 catalyst.

As shown in Fig. 10, three weight loss stages were observed for the Ru/HZSM-5 catalyst after usage [21]. The first weight loss stage was before 150 °C and the weight loss rate was 5%, which was attributed to water or other substances with low boiling points. The second weight loss stage at 150–300 °C (3.75% weight loss) was generally believed to be caused by “soft carbon”. The third stage at 300–700 °C (5.25% weight loss) corresponded to “hard carbon deposition”, accounting for 58.33% of the total carbon deposition. At the same time, the peak of the hard carbon curve could be wider and more complex as seen in the DSC curve, perhaps because there were more kinds of materials in the carbon deposition burned at high temperatures.

## Conclusions

In this investigation, a bifunctional Ru/HZSM-5 catalyst was prepared by ion exchange, chemical precipitation and hydrogen reduction. First, Ru particles were highly dispersed on the surface of HZSM-5 according to the XRD and TEM characterization results. According to the benzene alkylation reaction with methanol, the Ru/ZSM-5 catalyst could efficiently annihilate the MTO reaction and the formation of ethylbenzene. Meanwhile, it could effectively reduce the amount of trimethylbenzene and other byproducts. This excellent catalytic performance should be attributed to the large gap between the transgression energy value of Ru (74.5 eV) and the ionization potential value of benzene (8.5 eV). As a result, the benzene molecule was one of the most easily adsorbed species. and C_6_H_5_^+^ was formed on the surface of the catalyst, while methanol originally presents CH_3_O^−^ and H^+^. Therefore, the alkylation of benzene with methanol would react naturally. Compared with the HZSM-5 catalyst, the Ru/HZSM-5 catalyst exhibited excellent stability and high catalytic activity even after 150 h of usage. Our study pioneered a method to annihilate the MTO side reaction, which was of great importance to both theoretical research and potential industrial application.

## Data Availability

All data generated or analyzed during this study are included in this published article.
